# Corrigendum: Epigallocatechin gallate decreases plasma triglyceride, blood pressure, and serum kisspeptin in obese human subjects

**DOI:** 10.3389/ebm.2024.10179

**Published:** 2024-04-25

**Authors:** Saimai Chatree, Chantacha Sitticharoon, Pailin Maikaew, Kitchaya Pongwattanapakin, Issarawan Keadkraichaiwat, Malika Churintaraphan, Chanakarn Sripong, Rungnapa Sririwichitchai, Sompol Tapechum

**Affiliations:** Department of Physiology, Faculty of Medicine, Siriraj Hospital, Mahidol University, Bangkok, Thailand

**Keywords:** obesity, blood pressure, epigallocatechin gallate, human adipocytes, kisspeptin, plasma triglyceride

In the published article, there is an error in the Certificate of Ethical Approval Number in the **Ethics Statement**. The published certificate of approval number was mistakenly stated as “si344/2558(EC2).” The correct number for the Certificate of Ethical Approval Number is “Si 563/2015”.

The authors apologize for this error and state that this does not change the scientific conclusions of the article in any way.

